# Milk disrupts p53 and DNMT1, the guardians of the genome: implications for acne vulgaris and prostate cancer

**DOI:** 10.1186/s12986-017-0212-4

**Published:** 2017-08-15

**Authors:** Bodo C. Melnik

**Affiliations:** Department of Dermatology, Environmental Medicine and Health Theory, University of Osnabrück, Am Finkenhügel 7a, D-49076 Osnabrück, Germany

**Keywords:** Acne vulgaris, Cancer, Chromatin, DNA methyltransferase 1, Exosome, Gene expression, Milk, miRNAs, p53, Prostate cancer

## Abstract

There is accumulating evidence that milk shapes the postnatal metabolic environment of the newborn infant. Based on translational research, this perspective article provides a novel mechanistic link between milk intake and milk miRNA-regulated gene expression of the transcription factor p53 and DNA methyltransferase 1 (DNMT1), two guardians of the human genome, that control transcriptional activity, cell survival, and apoptosis. Major miRNAs of milk, especially miRNA-125b, directly target TP53 and complex p53-dependent gene regulatory networks. *TP53* regulates the expression of key genes involved in cell homeostasis such as *FOXO1*, *PTEN*, *SESN1*, *SESN2*, *AR*, *IGF1R, BAK1*, *BIRC5*, and *TNFSF10*. Nuclear interaction of p53 with DNMT1 controls gene silencing. The most abundant miRNA of milk and milk fat, miRNA-148a, directly targets DNMT1. Reduced DNMT1 expression further attenuates the activity of histone deacetylase 1 (HDAC1) involved in the regulation of chromatin structure and access to transcription. The presented milk-mediated miRNA-p53-DNMT1 pathway exemplified at the promoter regulation of survivin (*BIRC5*) provides a novel explanation for the epidemiological association between milk consumption and acne vulgaris and prostate cancer. Notably, p53- and DNMT1-targeting miRNAs of bovine and human milk survive pasteurization and share identical seed sequences, which theoretically allows the interaction of bovine miRNAs with the human genome. Persistent intake of milk-derived miRNAs that attenuate p53- and DNMT1 signaling of the human milk consumer may thus present an overlooked risk factor promoting acne vulgaris, prostate cancer, and other p53/DNMT1-related Western diseases. Therefore, bioactive miRNAs of commercial milk should be eliminated from the human food chain.

## Introduction

Milk is the postnatal nutrient and programming system of mammals, which promotes adequate growth and organ development. Growth and tissue maturation require enhanced gene expression, which is controlled by a host of genetic and epigenetic factors recently linked to complex interacting signal transduction pathways mediated by milk’s mTORC1-activating essential amino acids as well as milk exosome-derived miRNA signaling [1–8]. The transcription factor p53, designated as the guardian of the human genome [9], controls approximately 1/10th of human gene promoters that contain p53-binding sites and are thus p53 target genes [10, 11]. Dependent on the particular gene, p53 either promotes or inhibits gene expression [9–11]. High-confidence p53 target genes are involved in multiple cellular responses, including cell cycle arrest and apoptosis [11]. Recent evidence underlines that miRNAs control p53 expression [12]. Intriguingly, several miRNAs detected in human and bovine milk are known suppressors of *TP53*, the gene expressing p53. The very high sequence homology of human and bovine milk miRNAs [13], especially in their seed region governing their function, suggests that uptake of milk-derived miRNAs may compromise the delicate balance in the level of key gene regulatory miRNAs deregulating the consumer’s gene expression, which promotes pathophysiological processes such as acne vulgaris (Av) and prostate cancer (PCa). Thus, the question arose as to whether milk exosome-derived miRNAs affect the expression of p53 and p53-dependent gene regulation of the milk recipient such as the breastfed newborn infant and the regular consumer of cow’s milk. From a mechanistic point of view, an attenuation of p53-mediated cell cycle inhibition and apoptosis during the lactation period would facilitate cell cycle progression, growth and anabolism of the newborn infant. To achieve this goal, milk should interact with the p53-related gene regulatory network of the milk recipient. It is the intention of this perspective to provide translational evidence that milk miRNAs may be able to disrupt the homeostasis of p53 and DNMT1, the guardians of the human genome [9, 14]. This will be exemplified by a closer look to the two common Western diseases, acne vulgaris (Av) and prostate cancer (PCa), which both are closely related to increased consumption of commercial cow’s milk.

### Milk exosome-derived miRNAs resist intestinal degradation

Milk is the human body fluid that contains the highest amounts of RNAs and miRNAs [15]. These RNAs and miRNAs are predominantly secreted by mammary epithelial cells (MECs) and are transported via extracellular vesicles (exosomes) to fulfill their regulatory tasks in the complex setting of mammalian reproduction [2–7, 16]. There is increasing evidence that the specific encapsulation of milk miRNAs in exosomes (30–100 nm in diameter) and exosome-like vesicles (> 100 nm) confers protection against miRNA degradation and creates a long-distance signaling pathway for intestinal and vascular endothelial transport by endocytosis, a potential requirement for miRNA delivery to peripheral tissues [17–24]. It has recently been demonstrated that human milk exosomes and their miRNAs survive digestion in vitro and are taken up by human intestinal cells [25]. Moreover, trans-epithelial transport of bovine milk exosomal miRNAs across intestinal Caco-2 cell monolayers indicated their potential to cross the intestinal barrier. Cow milk exosomes protect their miRNAs against harsh digestive processes and enable their crossing of the intestinal barrier to reach the blood circulation for distant cellular effects [26]. Notably, there was no significant difference in the levels of miRNA-148a, miRNA-21 and miRNA-25 between in vitro digested exosomes and their respective undigested controls [26]. Consumption of commercial milk resulted in a dose-dependent increase of miRNA-29b in peripheral blood mononuclear cells of healthy adult human volunteers associated with corresponding changes in gene expression [27]. Recently, Manca et al. [28, 29] provided compelling evidence that orally administered fluorophore (DiR)-labeled cow milk exosomes are bioavailable in mice. Notably, a fraction of exosomes escaped re-packaging in the intestinal mucosa. Labeled bovine milk exosomes accumulated in liver and spleen of mice. Exo-GLOW Red-labeled RNA derived from cow milk exosomes has been detected in the brain, kidneys, lungs and livers of mice after oral administration of these exosomes. The authors concluded that the cargos of dietary exosomes are delivered to peripheral tissues [28, 29]. Thus, accumulating evidence underlines the bioavailability of orally administered bovine milk exosomes and their miRNA cargo, which survives gastrointestinal degradation, reaches the systemic circulation and modifies gene regulation in recipient cells of peripheral tissues. With the same intention, orally administered bovine milk-derived exosomes have been successfully used for systemic drug delivery to tumor-bearing mice [30, 31].

### Milk exosomes deliver TP53-targeting miRNAs

The expression of the p53 gene (*TP53*) is tightly regulated via transcriptional and post-translational modulations. Le et al. [32] demonstrated that miRNA-125b is a bona fide negative regulator of p53 in both zebrafish and humans. miRNA-125b-mediated down-regulation of p53 is strictly dependent on the binding of miRNA-125b to a miRNA response element in the 3′-untranslated region (3′ UTR) of *TP53* mRNA (Table 1). It has recently been shown that miRNA-125b directly represses 20 novel targets in the vast p53 network including both apoptosis regulators like BAK1, IGFBP3, ITCH, PUMA, PRKRA, TP53INP1, TP53, ZAC1, and also cell-cycle regulators like cyclin C, CDC25C, CDKN2C, EDN1, PPP1CA, SEL1L, respectively [32]. Notably, miRNA-125b regulation of p53 is conserved at the network level in all vertebrates [33]. Milk contains abundant miRNA-125b, which has been demonstrated in human [34], bovine [18, 35], and porcine milk exosomes [36], respectively. Further TP53 targeting miRNAs are miRNA-30d, miRNA-25, and miRNA-504 [37]. miRNA-25 and miRNA-30d directly target the 3′-UTR of *TP53* to down-regulate p53 protein levels and to reduce the expression of genes that are transcriptionally activated by p53 [37]. Notably, miRNA-30d has been detected as a major signature miRNA of mature raw and commercial milk of dairy cows [38]. miRNA-30d has also been found in porcine milk exosomes and in human milk [35, 39, 40]. In addition, miRNA-25-3p has been observed in human and porcine milk exosomes [34, 39].

**Table 1:**
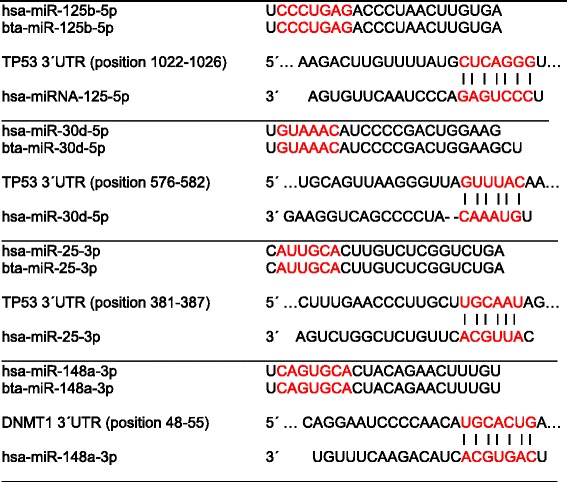
Illustration of human (hsa) and bovine (bta) mature and seed sequences of TP53- and DNMT1-targeting miRNAs (mirbase.org) with predicted base pairing regions marked in red (targetscan.org)

Remarkably, the mature and seed sequences of human and bovine miRNA-125b, miRNA-25, as well as miRNA-30d are identical (Table 1). This interspecies miRNA homology implies that milk miRNA-mediated p53 regulation may be a highly conserved archaic signaling pathway of mammals. These miRNAs target distinct 3’UTR regions of *TP53*.

### Milk-derived miRNAs and DNMT1 regulation

**Fig. 1:**
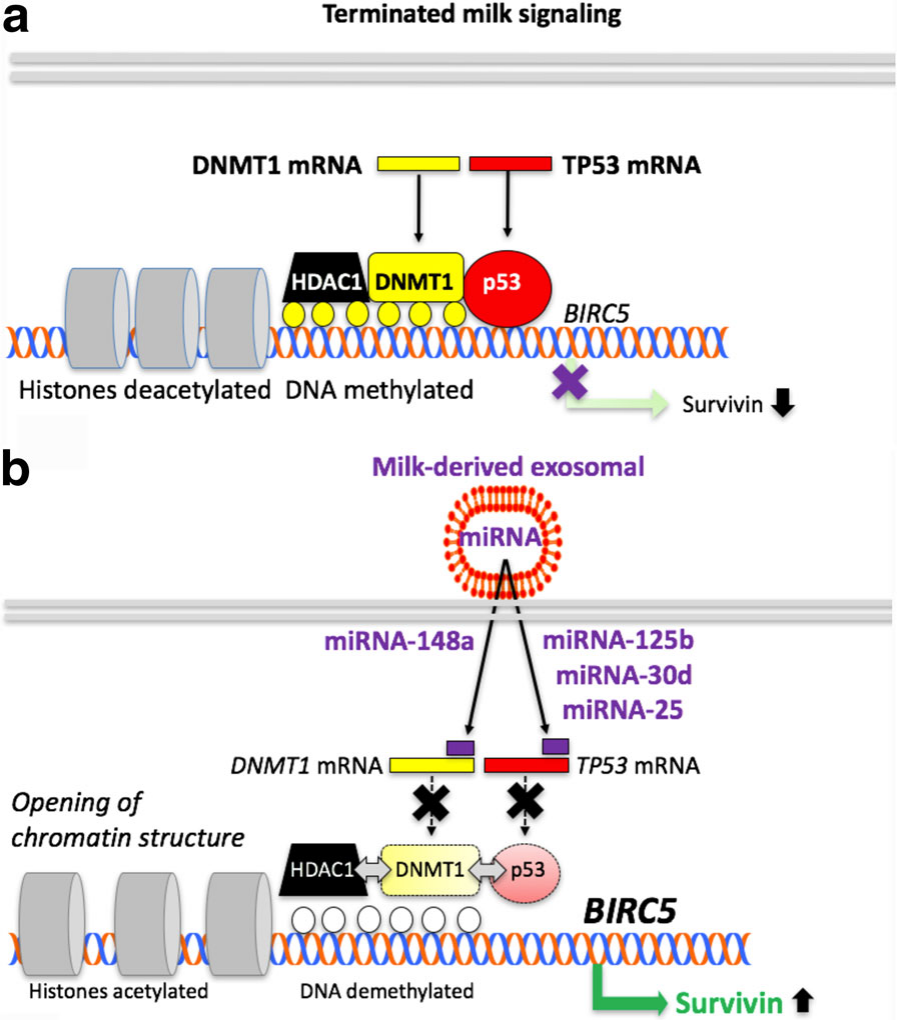
Illustration of milk microRNA signaling regulating the expression of *BIRC5*, the gene encoding the anti-apoptotic protein survivin. **a**. In the absence of milk-derived miRNAs, p53 suppresses survivin expression and attracts DNA methyltransferase 1 (DNMT1) to the *BIRC5* promoter, which results in DNA methylation (*yellow circles*, gene silencing). DNMT1 binding to histone deacetylase 1 (HDAC1) results in histone deacetylation and subsequent chromatin compaction. **b**. During milk consumption, milk exosomal microRNAs enter the recipient cells and down-regulate the expression of both TP53 and DNMT1 mRNA leading to increased survivin expression. DNMT1 and HDAC1 are removed from the *BIRC5* promoter resulting in DNA promoter demethylation and histone acetylation promoting gene expression and opening chromatin structure. Milk-derived p53- and DNMT1 targeting miRNAs thus operate as a switch regulating gene expression

Recent evidence underlines that DNA methylation is another “guardian of the genome” [41]. It is generally accepted that DNA hypomethylation destabilizes the genome [42]. DNA methylation is regulated by DNA methyltransferases [42]. The maintenance DNA methyltransferase (DNMT) 1 and the de novo methyltransferases DNMT3A and DNMT3B are all essential for mammalian development [42]. Thus, DNA methylation catalyzed by DNMTs plays an important role in maintaining genomic stability. Aberrant expression of DNMTs and disruption of DNA methylation patterns are closely associated with many forms of cancer [43–45]. The function of DNMT1 is tightly related to growth control [46]. DNMT1 is responsible for cytosine CpG methylation of DNA in mammals and has a role in gene silencing [42]. DNA methylation represses genes partly by recruitment of the methyl-CpG-binding protein MeCP2, which in turn recruits histone deacetylases [47]. DNMT1 is itself associated with histone deacetylase activity in vivo [47]. One of the known histone deacetylases, HDAC1, has the ability to bind directly to DNMT1 [47]. Thereby, DNMT1-mediated DNA methylation alters the chromatin state via HDAC activity [47]. The DNMT1 complex with HDAC1, Rb, and E2F1 represses transcription from E2F-responsive promoters [48] (Fig. 1).

Taken together, there is an intimate interaction between DNA methylation, histone deacetylase and sequence-specific DNA binding activity and the control of growth-regulatory pathways that are disrupted in nearly all cancer cells [48].

miRNA-148a is by far the most abundant miRNA detected in human milk, bovine colostrum and mature cow’s milk, porcine colostrum and mature porcine milk [25, 34, 38, 39, 49–51]. Furthermore, miRNA-148a is highly expressed in human and bovine milk fat [13, 50, 51] and has been detected in substantial amounts in bovine skim milk and human milk exosomes [13, 25, 34]. It is possible that milk fat globules (MFGs) of cow’s milk release miRNA-148a carried in crescent exosomes of MFGs [52], especially after the process of homogenization [50]. Golan-Gerstl et al. [13] recently demonstrated that miRNA-148a-3p represents the top one miRNA of pasteurized skim milk (16.09% of all miRNAs) and the top two miRNA of pasteurized milk fat (7.16%), respectively. These data correspond to the findings of Do et al. [53] who confirmed that miRNA-148a belongs to the most abundantly expressed miRNA of bovine milk since it accounts for more than 10% of the read counts in each stage of dairy cow lactation. miRNA-148a, miRNA-148b, and miRNA-152 are three members of the miRNA-148/152 family, which shares substantial homology in their seed sequences [54].

Wang et al. [55] reported that the expression of miRNA-152 significantly increased during lactation in MECs of dairy cows producing high quality milk compared to lower miRNA-152 levels in cows producing low quality milk. The forced expression of miRNA-152 in dairy cow MECs resulted in a marked reduction of DNMT1 at both the mRNA and protein levels [55]. Importantly, DNMT1 is a direct target of miRNA-148a [56]. The expression of DNMT1 is thus inversely related to the expression miRNA-148a and its family homolog miRNA-152 [57, 58]. Importantly, it has recently been shown that the expression of miRNA-148a of normal colon cells (CRL1831) and K562 leukemia cells increased after incubation with milk exosomes and the fat layer isolated from human milk [13]. The increased cellular expression of miRNA-148a was associated with a significant decrease in the expression of DNMT1 [13]. Lactogenic hormones such as prolactin induce cellular and extracellular miRNA-148a expression in bovine MECs [59]. Furthermore, miRNA-148a has been shown to induce milk triacylglycerol synthesis in goat MECs [60]. A recent study investigated genome-wide miRNA binding site variation between extinct wild aurochs and modern cattle and identified candidate miRNA-regulated domestication genes that enhance lactation performance including the *MIR148A* gene [61]. In fact, recent co-expression and network and pathway analyses identified bovine miRNA-148a as a major determinant enhancing milk yield [62].

miRNA-21 is another abundant miRNA of human and cow’s milk [38], which indirectly inhibits DNMT1 expression by targeting Ras guanyl nucleotide-releasing protein-1 (RASGRP1) [56].

It is of critical importance to mention that the mature and seed sequences of human and bovine miRNA-148a are identical (Table 1). miRNA-148a homology of DNMT1-targeting miRNA-148a is regarded as an ancestral epigenetic regulator in various mammalian species [13]. DNMT1 suppression appears to be a generalized signaling mechanism of milk capable of enhancing mammalian gene expression, a meaningful regulatory step for the growing mammal during the period of lactation but a critical gene-destabilizing system during long-term exposure.

### Cooperation between p53, DNMT1 and HDAC

**Fig. 2:**
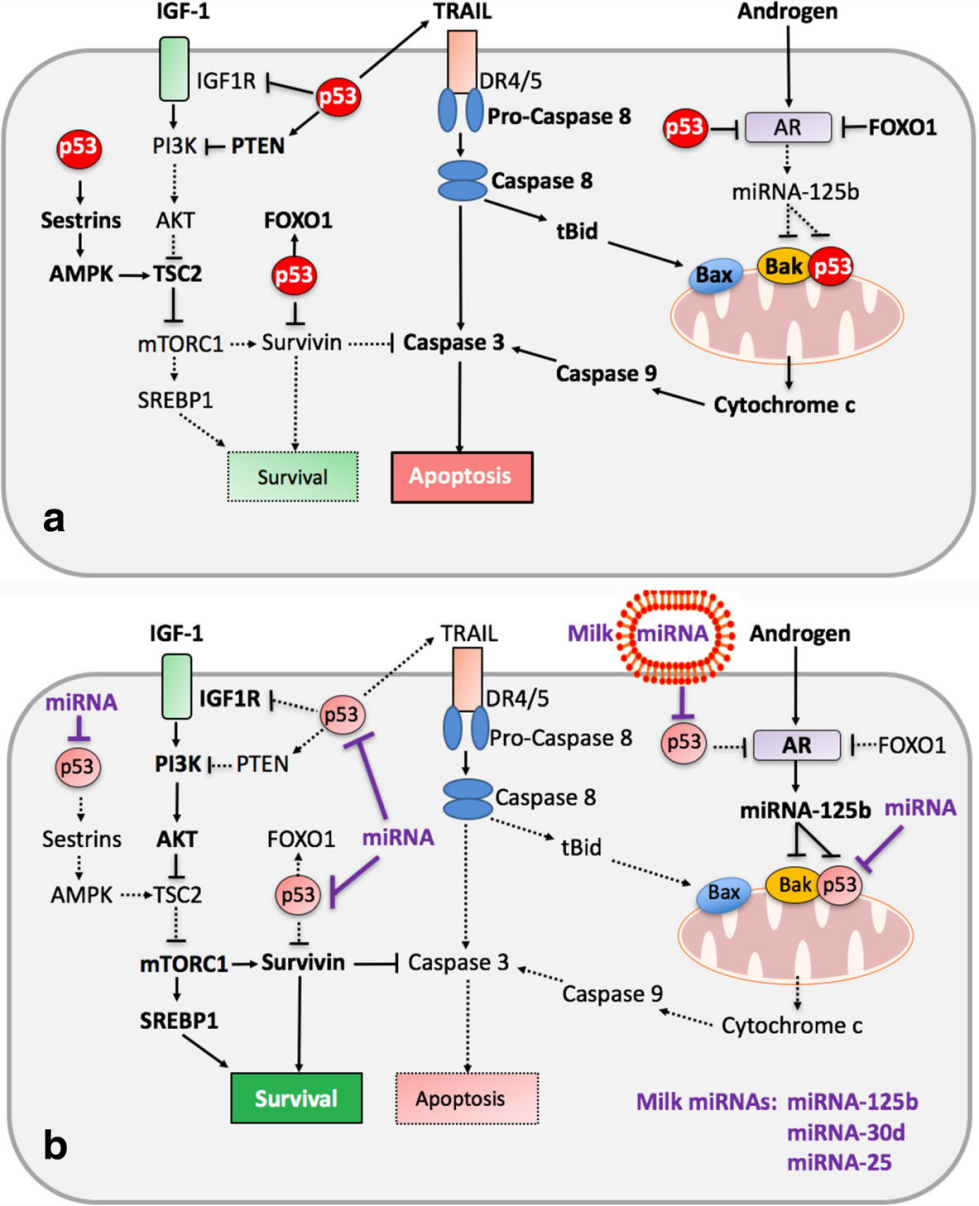
Working model illustrating the potential impact of milk exosome-derived miRNAs on the expression of p53. **a**. In the absence of milk miRNAs, p53 is abundantly expressed. p53 inhibits mechanistic target of rapamycin complex 1 (mTORC1) signaling and directly reduces the expression of IGF-1 receptor (IGF1R). p53 enhances sestrin1/2-mediated activation of AMK kinase (AMPK), a critical negative regulator of mTORC1. Furthermore, p53 induces the expression of FOXO1, which is a negative regulator of androgen receptor (AR). The expression of tumor necrosis factor-related apoptosis-inducing ligand (TRAIL) is upregulated by p53, which activates the extrinsic and intrinsic (mitochondrial) pathway of apoptosis. AR expression is negatively regulated by p53 resulting in decreased AR-mediated expression of miRNA-125b, which targets the pro-apoptotic proteins Bak and p53 at the mitochondrial membrane. This stimulates the interaction of p53 with Bak promoting the intrinsic pathway of apoptosis. **b**. During milk intake, milk exosomal miRNAs interrupt p53 signaling. This promotes the PI3K-AKT-mTORC1 pathway enhancing the expression of survivin, which is a negative regulator of caspase 3. Furthermore, milk miRNAs via attenuation of p53 and FOXO1 expression enhance AR signaling with subsequent upregulation of miRNA-125b. Increased miRNA-125b expression via AR signaling and milk miRNA-125b uptake down-regulate Bak-p53-interaction suppressing the intrinsic pathway of apoptosis. Thus, milk orchestrates both pro-survival and anti-apoptotic signaling, a most favorable constellation for the growing infant but a disastrous promoter of diseases in patients associated with disrupted p53 homeostasis such as acne vulgaris and prostate cancer

Cumulative work suggests that p53 is not a stand-alone regulator but participates in a complex gene regulatory network. DNMT1, a key regulator of epigenetics, is an integral part of this network. DNMT1 has been shown to physically interact and bind to p53 and co-localizes in the nucleus [63]. Upon p53 induction, a reporter construct containing the promoter of the anti-apoptotic gene survivin (*BIRC5*), which contains a natural p53 binding site, was methylated in wild type HCT116 cells, but not in DNMT1 null or p53 null cells. Endogenous survivin gene repression involves cooperation between DNMT1 and p53 (Fig. 1), which is relieved by introduction of DNMT1- or p53-specific small inhibitory RNA (siRNA) [63]. It is thus conceivable that milk exosome-derived miRNAs in a comparable manner to siRNAs target p53 and DNMT1 and activate survivin expression and other anti-apoptotic and growth promoting genes that are regulated in a p53-DNMT1-interacting fashion (Fig. 1). In contrast, activation of p53 down-regulates the anti-apoptotic protein survivin (Fig. 2) [64]. Survivin expression is absent in benign prostate specimens but high and associated with higher Gleason score in PCa [65]. DNMT1 is able to associate with histone deacetylase (HDAC) [10, 47], suggesting its involvement in transcriptional repression of chromatin by means of histone deacetylation [63]. DNMT1 activity via maintenance of the appropriate histone H3 modifications contributes to the preservation of the correct organization of large heterochromatic regions [64].

Milk miRNA-148a-mediated DNMT1 suppression may thus modify chromatin structure, unwinding chromatin to allow access to the DNA sequence and subsequent transcription, important regulatory events for the growing mammal. As discussed for the *BIRC5* promoter, p53 stabilizes the p53-DNMT1-HDAC1 complex (Fig. 1) [63]. DNMT1 potentially provides a key interaction via DNA methylation recruiting HDAC1 and acting as a bridge between nuclear p53 and chromatin, reinforcing a repressed chromatin state [66]. Milk miRNAs via targeting both p53 and DNMT1 would thus destabilize these key guardians of the human genome enhancing transcriptional activity for the restricted and privileged period of lactation (Fig. 1).

### Milk miRNAs survive pasteurization and homogenization

Pasteurization of milk has been introduced into modern dairy technology to limit bacterial growth but not to eliminate milk’s biological miRNA activity. There is recent evidence that substantial amounts of miRNAs of commercial cow’s milk resist pasteurization and homogenization [13, 35, 67]. Milk miRNAs such as miRNA-21 and miRNA-29b were found stable under different household storage conditions indicating that milk miRNAs could be available to the human milk consumer [27, 35, 67]. Notably, the vast majority of cow milk-derived miRNA-148a has been shown to survive pasteurization, homogenization, and the attacks of digestive enzymes in comparison to untreated cow’s milk [13, 26, 50]. In contrast, fermentation of cow’s milk by addition of probiotic bacteria resulted in a substantial loss of milk’s exosomal miRNAs exemplified by decreased recovery of miRNA-29b and miRNA-21 [23]. The authors discussed that bacterial attacks of exosome proteins deteriorates exosome membrane integrity, which may result in exosome miRNA degradation by miRNases secreted from microbes [23]. In comparison to pasteurized milk, fermented milk products such as yoghurt may thus exert diverse miRNA-dependent effects on human health. In fact, the EPIC-Interact Study (*n* = 340,234) demonstrated an inverse association of type 2 diabetes mellitus with a higher combined intake of fermented milk products compared to an increased association with unfermented milk [68]. With the widespread introduction of refrigeration technology in the early 1950’s, milk’s bioactive miRNAs unnoticeably entered the human food chain in a great and uncontrolled extent [6].

### The link between milk miRNA signaling, acne vulgaris and prostate cancer

There is accumulating evidence that milk consumption during adolescence is linked to the occurrence or aggravation of Av [69–80]. Recent evidence points to increased pro-survival and attenuated apoptotic signaling in the pathogenesis of Av [81, 82], which is therapeutically balanced by pro-apoptotic treatment with isotretinoin (*13-cis* retinoic acid, the prodrug of *all-trans* retinoic acid (ATRA) [83, 84]. One most important ATRA-responsive gene is p53 [85]. Increased insulin/IGF-1 signaling in Av activates the kinase AKT, which inactivates nuclear FoxO1 via AKT-mediated phosphorylation [86–88]. It is important to note that nuclear FoxO1, the transcription factor of starvation [89], suppresses the androgen receptor (AR), a critical acne-inducing pathway also involved in PCa [90–94]. FoxO1 is also a negative regulator of the oncogenic transcription factor *sterol regulatory element binding protein 1c* (SREBP1c) [95, 96], which plays an important role in excessive sebaceous lipogenesis [97], a hallmark of Av [76]. Remarkably, p53 stimulates the expression of *FOXO1* and phosphatase and tensin homolog (*PTEN*) [98]. PTEN antagonizes phosphoinositide 3-kinase (PI3K) and thus operates as a negative regulator of the IGF-1/PI3K/AKT signaling pathway that inactivates FoxO1 at the posttranslational level. Notably, weak p53 activity resulting from the single-nucleotide polymorphisms p53 G72C has been reported in the acne-associated SAPHO (synovitis, acne, pustulosis, hyperostosis, and osteitis) syndrome [99] (Fig. 2).

Therapeutic upregulation of the death receptor ligand *tumor necrosis factor-related apoptosis-inducing ligand* (TRAIL), which initiates apoptosis signaling has been identified as the primary mode of isotretinoin action in Av [83, 84]. TRAIL activation is a recent major therapeutic strategy in the treatment of PCa [100–103]. It is important to realize that p53 directly induces the expression of TRAIL [104]. There are two p53 DNA-binding sites in the human TRAIL promoter region [104]. Thus, milk-miRNA-mediated down-regulation of p53 expression may attenuate apoptotic TRAIL signaling implicated to play a crucial role in the pathogenesis of Av and cancerogenesis of PCa (Fig. 2).

It is of critical concern that daily milk consumption in adolescence (vs. less than daily) was associated with a 3.2-fold risk of advanced PCa [105]. These findings point to a link between milk consumption, Av and PCa. In fact, an increased risk of PCa has been observed in men who suffered from more severe Av, which required oral tetracycline treatment [106]. Notably, increased IGF-1 and AR-mediated signaling with increased expression of survivin are related to both Av and PCa [88, 106–109]. Among patients diagnosed with localized PCa, compared to men who consumed <1 servings/day of high-fat milk, those who drank ≥3 servings/day had an increased hazard of PCa mortality [110]. Men with the highest versus lowest intake of whole milk were at an increased risk of progression among participants in the *Health Professionals Follow-Up Study* [111]. In accordance, whole milk intake but not total dairy protein intake has been associated with PCa-specific mortality among U.S. male physicians [112]. A recent comprehensive meta-analysis of 11 population-based cohort studies involving 778,929 individuals confirmed the association between milk consumption and PCa [113]. Milk-induced Av and milk-driven PCa are both associated with increased mTORC1 signaling [88, 114–117]. Serum levels of IGF-1, a key stimulator of mTORC1 activity [118–125], increase during milk intake [124, 125]. Enhanced IGF-1 signaling has been related with the pathology of both Av [109, 126–129] and PCa [130], respectively. A recent meta-analysis substantiated a significant association between milk consumption and increased IGF-1 serum levels in patients with PCa [130]. The addition of milk to LNCaP cells in culture enhanced PCa cell proliferation by 30% [131]. It is important to emphasize that IGF-1/mTORC1 signaling is negatively controlled by p53. p53 induces the expression of sestrin1 and 2 [132, 133], which activate AMP kinase (AMPK), a pivotal negative regulator of mTORC1. Furthermore, p53 controls IGF-1-mediated activation of mTORC1 via p53-mediated reduction of IGF1 receptor (*IGF1R*) expression (Fig. 2) [134, 135].

Loss-of-function mutations of p53 are associated with a multitude of human cancers including PCa [136–139]. PCa specimen staining for p53 was inversely correlated with the age of patients with PCa [140]. Notably, Spyridonidis et al. [141] reported PCa in a patient with SAPHO syndrome. A persistent down-regulation of p53 activity via p53 targeting milk-derived miRNAs may thus enhance PCa progression. Remarkably, a Swedish cohort study reported a relationship between daily milk intake and overall mortality [142]. In this study, milk consumption correlated with increased serum levels of interleukin-6 (IL-6) [142]. Intriguingly, p53 has been identified as a key suppressor of IL-6 and plays a pivotal role in suppressing inflammation and oxidative stress [143, 144]. Increased IL-6 expression has been detected in PCa specimens and has been related to PCa carcinogenesis [145]. IL-6 is also a major pro-inflammatory mediator that is up-regulated in the skin of patients with Av (Table 2) [146, 147] .

**Table 2:** p53-dependent signaling pathways of milk, acne, and prostate cancer

Targets	Milk	Acne	PCa	References
IGF-1/IGF1R	+	+	+	109,123–130
FOXO1	?	−	−	86–88, 90–94
Androgen/AR	+	+	+	108,145,146
mTORC1	+	+	+	1114–117
Survivin	?	+	+	107–109
TRAIL	?	−	−	83,84,100–103
IL-6	+	+	+	141,144–146
miRNA-125b	+	?	+	15,27,32,33,29,180
miRNA-25	+	?	+	27,36,180

*IGF-1* insulin-like growth factor 1, *IGF1R* IGF1 receptor, *FOXO1* forkhead box O1, *AR* androgen receptor, *TRAIL* tumor necrosis factor-associated apoptosis-inducing ligand, *IL-6* interleukin 6

The etiology of benign prostatic hyperplasia (BPH) and prostatic neoplasia, which can progress to PCa, is androgen-dependent, and reduction/obliteration of androgen action in the prostate has been the therapy of choice for BPH and PCa [148]. A recent study demonstrated that cases of PCa exhibited a higher staining intensity for AR when compared with BPH [149]. Remarkably, AR is a direct negatively regulated target of p53 [150, 151]. Due to persistent pregnancy of dairy cows, commercial milk contains increased amounts of androgen-precursors recently related to pathogenesis of Av [152]. Milk-derived androgens as well as milk-derived miRNAs suppressing p53 may both enhance AR-dependent signaling.

There is accumulating evidence derived from systematic meta-analyses that raised serum IGF-1 levels are associated with increased risk of PCa [153–157]. At present, epidemiologists focus on the role of milk-induced IGF-1 signaling in PCa and have demonstrated a relationship between milk consumption, increased serum IGF-1 levels and increased risk of PCa [130]. Unfortunately, these IGF-1 centered studies did not consider the potential influence of milk-derived miRNAs that are highly bioactive components of whole milk.

Reduced expression of DNMT1 plays an important role in the induction of epithelial-mesenchymal transition (EMT) and cancer stem cell (CSC) phenotype in PCa cells, which has been associated with tumorigenesis and metastasis [158]. Reduction of DNMT1 by 5-azacytidine (5-Aza) promoted EMT induction as well as CSCs in vitro [158]. Thus, reduced DNMT1 expression via continued uptake of milk-derived DNMT1-tageting miRNA-148a may promote EMT and the CSC phenotype facilitating PCa progression [158]. The absence of miRNA-148a and related DNMT1 signaling may explain why miRNA-deficient milk protein powder did not affect prostate tumor progression in two mouse models of benign and neoplastic lesions [159], whereas commercial milk including bioactive miRNAs added to PCa cells in culture significantly promoted cell proliferation [131].

### Milk-miRNA-125b counteracts antiandrogen therapy of prostate cancer

Most clinical PCa specimens overexpress miRNA-125b, which is regarded as an oncogene of PCa [160, 161]. miRNA-125b directly targets three key pro-apoptotic genes: *TP53*, *BBC3* (Puma), and *BAK1*. Increasing the abundance of miRNA-125b results in a dramatic decrease in the levels of these apoptosis effectors in PCa cells [162]. Remarkably, androgen stimulation of PCa cells via AR up-regulates the expression of miRNA-125b (Fig. 2), thus reduces the expression of these pro-apoptotic proteins [160, 161]. Bak1 expression was detected in 77.5% of primary and untreated localized PCa, but only in 33% of hormone-refractory PCa [163]. Therefore, downregulation of *BAK1* by miRNA-125b may contribute to disease progression and resistance to treatment in PCa [160, 161]. miRNA-125b was found to have the ability of rendering LNCaP cells resistant to androgen withdrawal [161]. Milk consumption during antiandrogen therapy of PCa via transfer of milk-derived miRNA-125b may counteract antiandrogen-induced suppression of miRNA-125b thereby reducing the level of PCa cell apoptosis.

Upregulated p53 interacts with the pro-apoptotic mitochondrial membrane protein Bak, which causes oligomerization of Bak and release of cytochrome c from mitochondria. Evidence has been provided that Bak may serve as a mitochondrial receptor for p53 [164]. Notably, the p53-Bak complex coincides with the loss of interaction between Bak and the anti-apoptotic Bcl2-family member Mcl1. Thus, p53 and Mcl1 have opposing effects on mitochondrial apoptosis by interacting with, and modulating the activity of the death effector Bak [165, 166].

Milk-miRNAs targeting p53 may thus attenuate the p53-TRAIL-mediated extrinsic pathway of apoptosis and the p53-Bak-regulated intrinsic pathway of apoptosis promoting pro-survival signaling (Fig. 2). Remarkably, IGF binding protein 1 (IGFBP1) has been reported to increase during cow milk consumption [167]. IGFBP1 via binding to BAK impairs the formation of the pro-apoptotic p53/Bak complex [168].

## Conclusions

Milk, the postnatal nutrient and signaling system of mammals, promotes growth and lifelong metabolic and immunological programming [169–172]. Milk’s miRNAs provide an “ancestral maternal language” that shapes the genome of the breastfed infant [1–7]. Presented translational evidence and mechanistic plausibility allow the prediction that milk-derived miRNAs attenuate the guardians of the genome, p53 and DNMT1, and their complex interacting regulatory network regulating gene expression and chromatin structure (Fig. 1). We have to keep in mind that under physiological conditions milk-dependent miRNA-mediated nutrigenomic effects are restricted to the lactation period, which is terminated after weaning in all mammals. In modern humans, however, technical interventions such as pasteurization and refrigerated storage of milk miRNAs allows lifelong exposure to milk’s archaic miRNA-orchestrated signaling machinery that persistently disturbs p53- and DNMT-dependent gene regulation of the milk recipient. Milk-derived miRNA-125b and androgen-stimulated miRNA-125b thus operate synergistically as a natural doping system.

Genetic and epigenetic selection of dairy cows intended to increase lactation performance and milk yield further enhances the expression of lactation-promoting miRNAs such as miRNA-148a [50, 59]. These miRNAs via milk exosome transfer finally target p53 and DNMTs of the milk consumer. Enhanced dairy cow lactation performance is thus an unnoticed risk factor that further increases the miRNA burden for the human milk consumer [6]. Persistent and increased milk miRNA-mediated down-regulation of p53 and DNMT1 most likely enhances the risk of diseases of civilization such as are Av and PCa [173, 174]. Strikingly, Av and PCa share common milk-dependent signal transduction pathways (Fig. 2). In this regard, it is a well-grounded recommendation for adolescents and adults to avoid the intake of pasteurized whole or skim milk, which delivers bioactive miRNAs that modify the human genome. Evidence in support of the bioavailability of miRNAs encapsulated in dietary exosomes [2–8] outweighs studies produced by scholars doubting that this phenomenon is real [175–177]. These studies have been disputed due to several technical or methodological pitfalls discussed elsewhere [8, 21, 178, 179]. Nevertheless, some important issues need more detailed future studies: 1) Direct evidence is still missing that shows the uptake of milk exosome-derived miRNAs in peripheral cells of the milk recipient. 2) It is not known with certainty that milk-derived miRNAs reach sufficient intracellular concentrations modifying gene regulation. 3) Although accumulating evidence underlines the uptake of milk exosomal miRNAs by intestinal cells and vascular epithelial cells [17–22], no direct evidence of selective milk miRNA uptake by other organs such as the sebaceous or prostate gland has yet been provided. However, it is of critical concern, that today’s milk contains and transfers oncogenic miRNAs involved in the cancerogenesis of PCa such as miRNA-21, miRNA-25, and miRNA-125b [180]. It is thus advisable to inactivate exosomal milk-derived miRNAs either via ultraheat or microwave treatment, ultra-sonication, or fermentation [6, 20, 46, 60, 68] in order to prevent an unperceived hazardous contamination of the human food chain with oncogenic biosimilar miRNAs of another mammalian species such as *Bos taurus*.

## Abbreviations

AMPK: AMP-activated kinase
Av: Acne vulgaris
BPH: Benign prostatic hyperplasia
CSC: Cancer stem cell
DNMT: DNA methyltransferase
DR: Death receptor
EMT: Epithelial-mesenchymal transition
FOXO1: Forkhead box O1
HDAC: Histone deacetylase
IGF-1: Insulin-like growth factor-1
IGF1R: IGF1 receptor
MEC: Mammary epithelial cells
MFG: Milk fat globule
miRNA: Micro ribonucleic acid
mTORC1: Mechanistic target of rapamycin complex 1
PCa: Prostate cancer
PI3K: Phosphoinositide-3 kinase
PTEN: Phosphatase and tensin homolog
SREBP1: Sterol regulatory element binding protein 1
TRAIL: Tumor necrosis factor-related apoptosis-inducing ligand
TSC2: Tuberin
UTR: Untranslated region
